# Micromagnetic Simulations of Fe and Ni Nanodot Arrays Surrounded by Magnetic or Non-Magnetic Matrices

**DOI:** 10.3390/nano11020349

**Published:** 2021-02-01

**Authors:** Devika Sudsom, Andrea Ehrmann

**Affiliations:** Faculty of Engineering and Mathematics, Bielefeld University of Applied Sciences, 33619 Bielefeld, Germany; donlawat.sudsom@fh-bielefeld.de

**Keywords:** micromagnetic simulation, OOMMF, nanodots, antidots, array, spintronics

## Abstract

Combining clusters of magnetic materials with a matrix of other magnetic materials is very interesting for basic research because new, possibly technologically applicable magnetic properties or magnetization reversal processes may be found. Here we report on different arrays combining iron and nickel, for example, by surrounding circular nanodots of one material with a matrix of the other or by combining iron and nickel nanodots in air. Micromagnetic simulations were performed using the OOMMF (Object Oriented MicroMagnetic Framework). Our results show that magnetization reversal processes are strongly influenced by neighboring nanodots and the magnetic matrix by which the nanodots are surrounded, respectively, which becomes macroscopically visible by several steps along the slopes of the hysteresis loops. Such material combinations allow for preparing quaternary memory systems, and are thus highly relevant for applications in data storage and processing.

## 1. Introduction

The magnetic properties and magnetization reversal of single nanoparticles or nanoparticle arrays are defined by the dimensions and shapes of the nanoparticles, as well as by their material. This interplay between magneto-crystalline and shape anisotropy can lead to unexpected and technologically applicable effects [1,2]. Round and square nanodots, nanodot arrays, and antidot arrays are often examined since they allow for building vortices with reduced in-plane stray fields [3,4,5,6,7,8].

On the other hand, combining different materials either in the form of thin layer stacks or in one plane offers new possibilities of controlling magnetization reversal processes. Most recently, Salaheldeen et al. investigated the overall anisotropies in magnetic hard/soft bilayer antidot arrays and found a crossover from magnetic in-plane to out-of-plane anisotropy due to the interface exchange coupling between the hard Co and the soft Py layer [9]. Verba et al. also coupled soft ferromagnetic thin films and nanodots to a hard magnetic antidot matrix, in this way allowing for tuning the helicity of a vortex or skyrmion in this material [10]. Negusse and Williams reported on exchange-spring materials, based on combining soft and hard magnetic layers [11]. Similar behavior was found earlier by Fullerton et al. [12,13], while Coey and Skomski aimed at finding material combinations with high energy products in their previous studies [14,15]. More recently, Sawicki et al. prepared epitaxial DyFe_2_-YFe_2_ superlattices with increased coercivity and exchange spring behavior [16]. Kim et al. prepared soft/hard magnetic composite fibers by electrospinning, combining iron oxide (soft) and cobalt ferrite (hard) magnetic materials, and found a higher saturation magnetization and coercivity than in pure CoFe_2_O_4_ [17]. Similarly, Zhang et al. prepared CoFe_2_O_4_/La_0.7_Sr_0.3_MnO_3_ soft/hard composites and found different saturation magnetization for different mixing ratios [18].

In our group, previously different in-plane combinations of hard/soft magnetic materials were examined, such as bow-tie structures, double-wedges, and semi-squares [19,20]. Combining the approaches of nanodot/antidot arrays and soft/hard magnetic materials, here we report on magnetically hard nanodots surrounded by a magnetically soft matrix and vice versa, and compare both situations with a pure magnetic hard nanodot array as well as with a mixed array. Our results show clearly different coercive fields and hysteresis loop shapes due to combining both magnetic materials in a nanodot/antidot structure. In addition, partly unexpected magnetization reversal processes occur. This paper is an extended version of [21].

## 2. Materials and Methods

**Figure 1:**
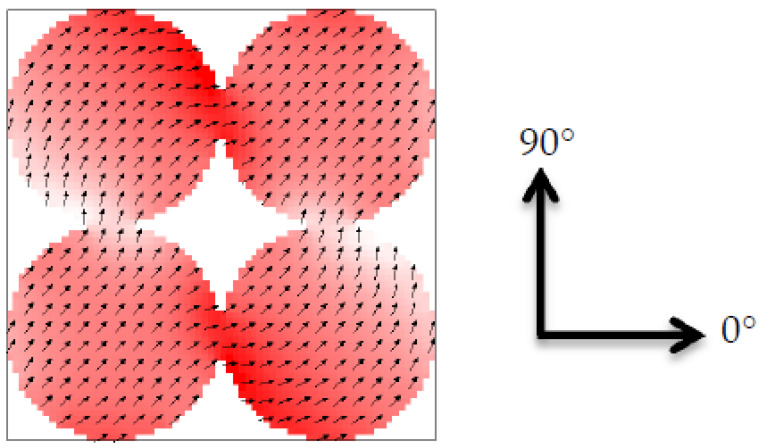
Sketch of the nanodot arrays under examination consisting of four touching nanodots (diameter 105 nm), surrounded by air or the other magnetic material, as well as the orientation of the external magnetic field angles. The color is defined by the magnetization pointing to the right side shown in red, magnetization to the left side depicted in blue, and magnetization pointing to the top or bottom in white.

Micromagnetic simulations were performed using the micromagnetic simulator OOMMF (Object Oriented MicroMagnetic Framework) [22] by dynamically solving the Landau–Lifshitz–Gilbert equation on a mesh built by finite differences [23]. To prepare bi-material maps, the function ImageAtlas was used, defining material positions by image files. Material parameters for iron (Fe) and nickel (Ni) were chosen in agreement with average literature values: magnetization at saturation M_S,Fe_ = 1700·10^3^ A/m (M_S,Ni_ = 490·10^3^ A/m), exchange constant A_Fe_ = 21·10^−12^ J/m (A_Ni_ = 9·10^−12^ J/m), and magneto-crystalline anisotropy constant K_1,Fe_ = 48·10^3^ J/m³ (K_1,Ni_ = −5.7·10^3^ J/m³). Average values between both materials were used to define the borders (M_S,border_ = 1095·10^3^ A/m, A_Border_ = 15·10^−12^ J/m K_1,border_ = 21.15·10^3^ J/m³) [24,25]; alternatively, the borders can be defined by one of the material properties [26]. To take into account the common production methods of such nanostructures by sputtering, random crystallographic orientations were chosen in each cubic grain of diameter 3 nm. Setting the Gilbert damping constant to α = 0.5 results in the simulation of a quasistatic case. Simulations were performed by sweeping the external magnetic field from positive to negative saturation and back. Investigating the transverse magnetization components M_T_ in addition to the longitudinal hysteresis loops M_L_ ensures that minor loops are avoided. The results of field sweeps along 30°, 45°, and 60° (cf. Figure 1) are shown. Simulations directly along the symmetry axes (i.e., along 0° and 90°) were avoided because such simulations tend to show arbitrary magnetization reversal, depending on smallest symmetry breaking effects, and are thus not reliable. Simulations along 30° and 60° should actually be identical; however, due to the aforementioned random crystallographic orientations, comparing them offers the opportunity to evaluate the possible modifications due to different crystallographic orientations.

The nanodot areas under examination are sketched in Figure 1. Four magnetic dots of diameter 105 nm are surrounded by a square of side length 210 nm. Outside the dots, there is either air or the second magnetic material, in this way forming an antidot array. The system height is 15 nm. No periodic boundary conditions were applied in order to avoid artificial interference effects [27]; instead, this simulation is a first extension of the common single nanodot which will be extended to larger arrays in the near future.

## 3. Results and Discussion

**Figure 2:**
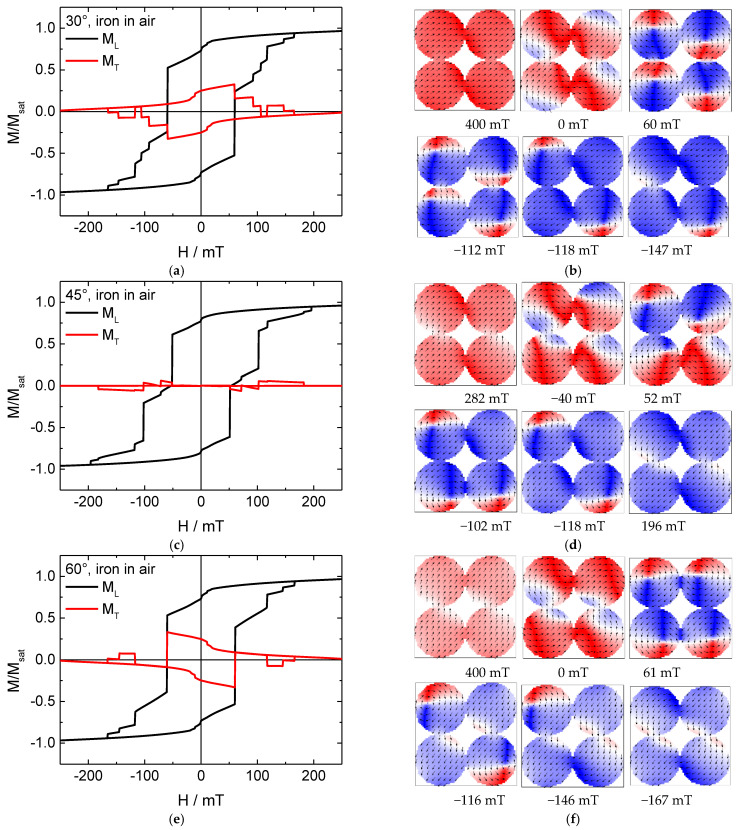
(**a**,**c**,**e**) Hysteresis loops and (**b**,**d**,**f**) snapshots of the magnetization reversal from positive to negative saturation for a pure iron nanodot array in air, simulated for the angles depicted in the graphs.

To start with a common structure, Figure 2 depicts longitudinal and transverse hysteresis loops M_L_ and M_T_, respectively, as well as snapshots during magnetization reversal from positive to negative saturation of the system consisting of four iron nanodots in air.

The longitudinal hysteresis loops of all angles first show a reduction of the magnetization, similar to the system described in [16], followed by several steps after passing the coercive field. The transverse loop additionally shows an open area around 0 mT, as is the case in Stoner–Wohlfarth magnetization reversal. Here, however, the Stoner–Wohlfarth-like behavior (i.e., magnetization reversal via coherent rotation of the magnetization) is strongly superposed by several jumps and steps, correlated to the formation and finally annihilation of vortices in the single nanodots.

For the middle angle of 45°, as Figure 2d clearly shows, even in this apparently simple system, magnetization reversal follows a complex path. While all nanodots seem to be well-aligned during saturation at first glance, a deeper look reveals some differences near the touching points between neighboring dots where the influence of the shape anisotropy is reduced due to the touching next dot. These differences become stronger for a reduced external magnetic state until a first dot switches into a vortex state, followed by the others one after the other. Then, these vortex states switch into negative saturation, again not simultaneously, but subsequently. All the steps in the resulting hysteresis loop (Figure 2c) correspond to switching one of the nanodots, similar to strongly extended Barkhausen jumps.

For the angles of 30° and 60°, the situation is slightly different. In both these cases, a situation is visible in which all nanodots are in a vortex state (Figure 2b,f). Interestingly, for an angle of 30°, both left and both right nanoparticles show identical chiralities (i.e., rotational orientations of the magnetization around the vortex core), while both lower and both upper nanoparticles show identical chiralities in the case of a 60° orientation. This means that magnetization orientation in the touching points of “horizontally” neighboring nanodots is identical in the case of 30° orientation, while this is valid for “vertically” neighboring nanodots for the 60° orientation. 

Apparently, for such four-nanodot clusters, the energetically favorable vortex state is defined by a superposition of reduction of domain walls (i.e., neighboring nanodots having identical magnetization orientation in the touching area, leading to all vortices having identical chirality) and reduction of external stray fields (i.e., neighboring nanodots having opposite chirality).

It should be mentioned that hysteresis loops of single Fe nanodots with similar dimensions or nanodot arrays show coercivities between approximately 0 mT in case of vortex states up to nearly 100 mT [4,8,28,29]; the values shown here are well within these borders.

**Figure 3:**
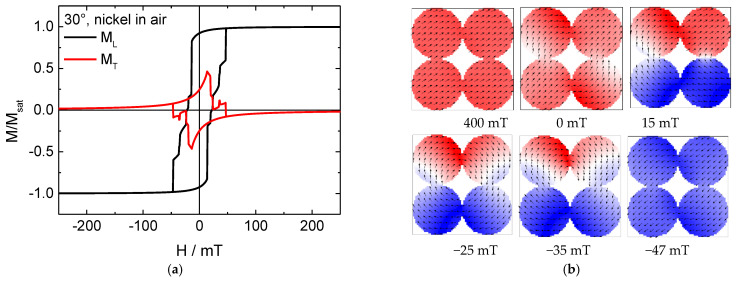

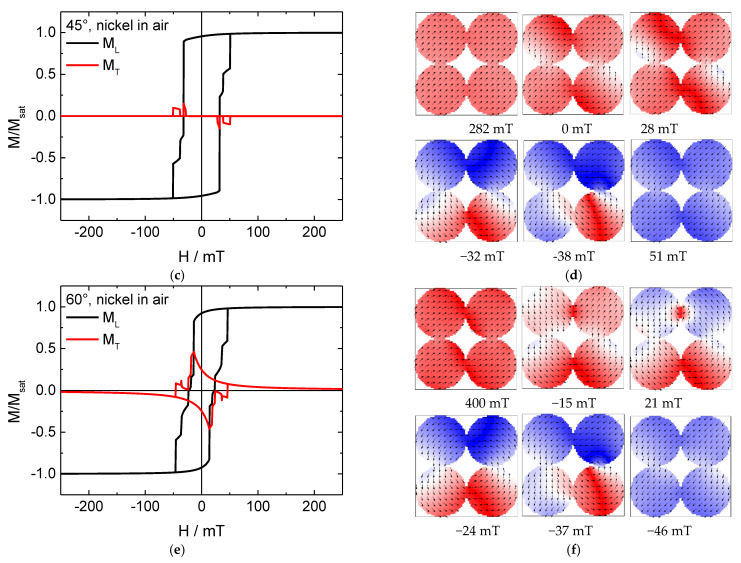
(**a**,**c**,**e**) Hysteresis loop and (**b**,**d**,**f**) snapshots of the magnetization reversal from positive to negative saturation for a pure nickel nanodot array in air, simulated for the angles depicted in the graphs.

The same situation simulated for pure Ni nanodots is depicted in Figure 3. Again, starting with the middle orientation of 45°, imagining all four nanodots as one large particle, magnetization reversal switches from an onion state in the first three snapshots to something similar to a vortex state in the fourth snapshot, with the magnetization rotating along all four dots, in this way creating a sort of vortex without a core. In the fifth snapshot, this already begins to change, before negative saturation is finally reached with a state similar to an overall onion state (Figure 3d).

For angles of 30° and 60°, first, a large horseshoe-like state is formed over all nanoparticles, until again a large vortex-like state comprising all four nanodots is reached until finally negative saturation is reached. For these angles, the transverse magnetization loops are again similar to Stoner–Wohlfarth magnetization reversal, again superposed by some steps which occur here due to domain wall processes.

The smaller coercivities found here for the Ni nanodot array are at the upper border of typical values found in the literature, which are in the range of 15–30 mT for Ni nanodot arrays of similar dimensions [30,31,32].

**Figure 4:**
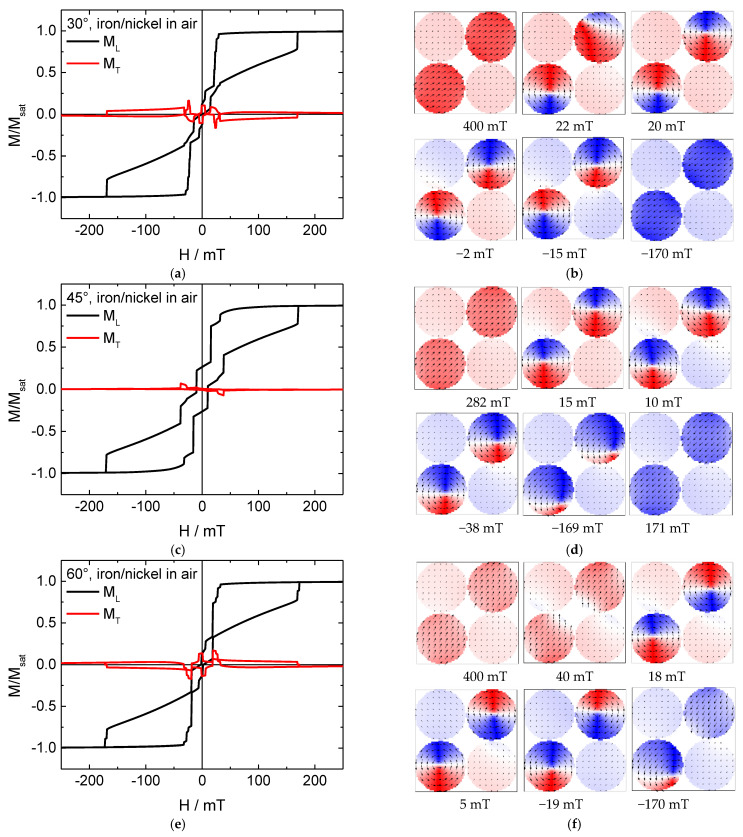
(**a**,**c**,**e**) Hysteresis loop and (**b**,**d**,**f**) snapshots of the magnetization reversal from positive to negative saturation for a mixed iron (upper right/lower left)/nickel (upper left/lower right) nanodot array in air.

Figure 4 shows hysteresis loops and snapshots of the magnetization reversal for a system combining iron and nickel nanodots in air. Interestingly, here the Fe nanodots can unambiguously switch into a vortex state which is kept during a large field range (e.g., from approx. +15 to −170 mT in the 45° orientation). The steps in this area are correlated with the first and the second nickel nanodot fully switching their magnetization.

This already shows that combining materials with relatively weak and significantly stronger anisotropies may lead to new, sometimes unexpected, magnetization reversal processes that can be used for new applications. It should be mentioned that no sign of coherent magnetization reversal is visible in the transverse magnetization loops.

Slight differences between the hysteresis loops simulated at 30° and 60° can be attributed to the random crystallographic orientations, chosen arbitrarily at the beginning of each simulation, as mentioned in Section 2.

**Figure 5:**
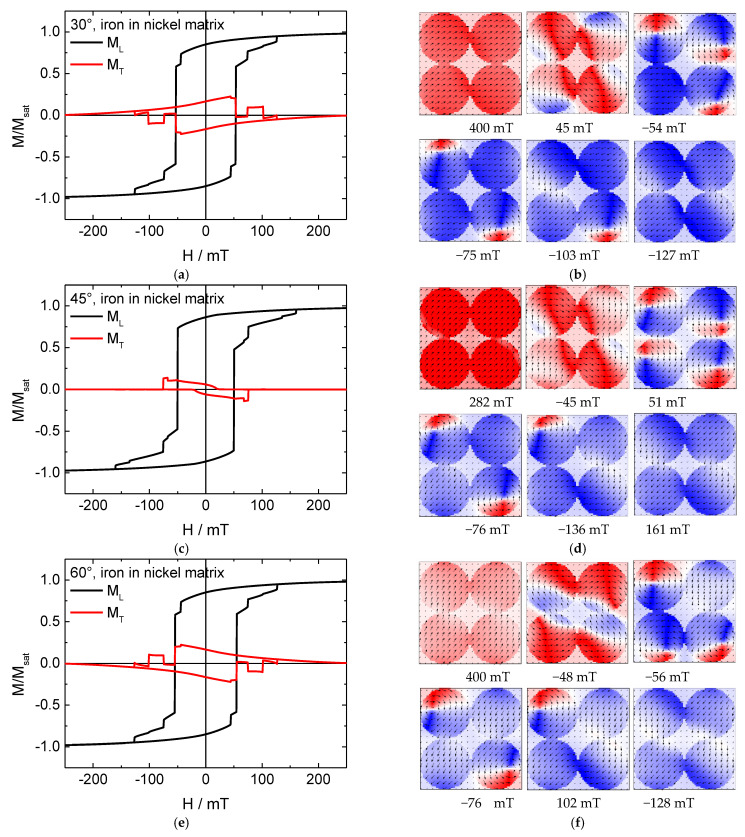
(**a**,**c**,**e**) Hysteresis loops and (**b**,**d**,**f**) snapshots of the magnetization of the reversal from positive to negative saturation for a pure iron nanodot array in a nickel matrix, simulated for the angles depicted in the graphs.

Next, the impact of a Ni matrix in which the Fe nanodots are surrounded was tested. The results are depicted in Figure 5. Comparing both longitudinal hysteresis loops shows significantly fewer steps for the Fe/Ni composite for all angles under examination, indicating a more consistent reversal throughout the whole system. The snapshots of the magnetization reversal at an angle of 45° (Figure 5d) indeed show an abrupt switching of all four Fe nanodots at the same time, corresponding to the main jump in the curve, while the two smaller steps are related to the next reversal into negative saturation by two of the nanodots, followed by the others.

For an angle of 30°, on the other hand, there is no step visible, with all four dots being in a vortex state simultaneously. For all angles, however, a partly coherent magnetization reversal is visible by the open transverse hysteresis loop around 0 mT.

Apparently, surrounding the iron nanodots with the nickel matrix stabilizes the magnetization reversal process and makes it less arbitrary, which is an important factor for the possible technological application of magnetic nanodots, and especially the vortex states available in them, for many dimensions in storage media.

**Figure 6:**
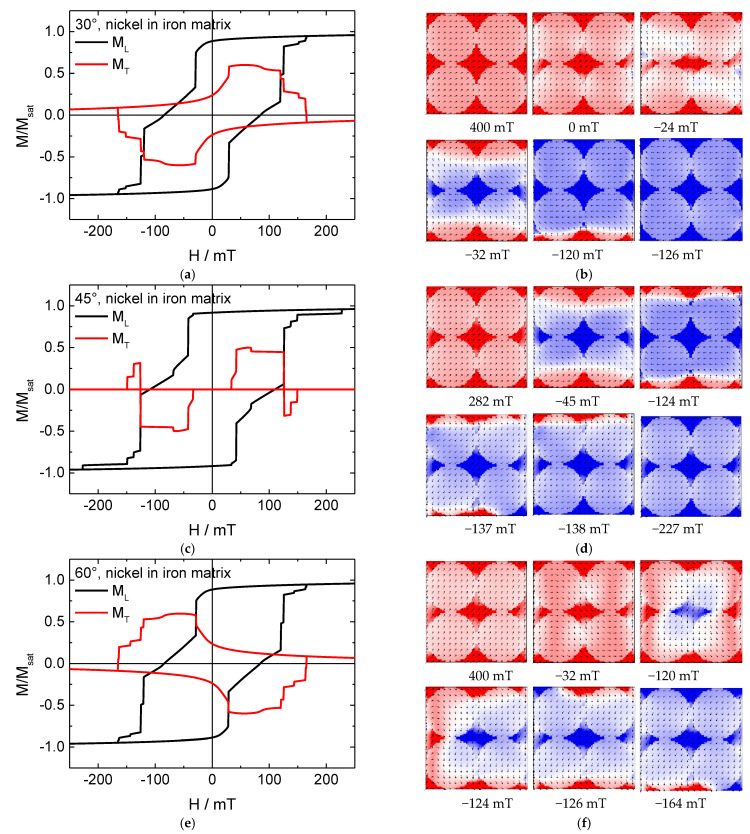
(**a**,**c**,**e**) Hysteresis loops and (**b**,**d**,**f**) snapshots of the magnetization reversal from positive to negative saturation for a pure nickel nanodot array in an iron matrix, simulated for the angles depicted in the graphs.

Testing the reversed matrix, Figure 6 depicts hysteresis loops and magnetization reversal snapshots from positive to negative saturation of nickel nanodots in an iron matrix. 

Here, the behavior is completely different. In the hysteresis loops simulated for 45° (Figure 6c), there are strong jumps in addition to several smaller ones, combined with a large transverse magnetization component which even changes signs on either side of the loop. In the systems with Fe nanodots, this energetically unfavorable configuration of a large fraction of the magnetization being perpendicular to the external magnetic field could be avoided by building vortex states, which is not usual in materials like nickel or permalloy with their small magneto-crystalline anisotropies [15]. Indeed, magnetization reversal snapshots show no vortices here, either in the nickel nanodots or in the iron antidot matrix where vortices are impeded by the shape.

Instead, domain walls are formed, preferably inside the nickel nanodots due to their small anisotropy, and only at the beginning and the end of the magnetization reversal process, these domain walls are partly located inside the Fe antidot matrix.

For angles of 30° and 60°, respectively, large, broad transverse magnetization components are found, connected with long steps in the longitudinal magnetization components. These Stoner–Wohlfarth-like transverse hysteresis loops are correlated with coherent rotation of the magnetization in large areas of the specimens, preceded and followed by jumps of the magnetization in parts of the structures. 

While in this approach of surrounding Ni with an Fe matrix, the boundaries of the model were set next to the nanodots, our first results suggest that a more interesting system can possibly be created by allowing iron bridges between the nickel nanodots as well as along the borders of the system.

## 4. Conclusions

Arrays of hard/soft magnetic material nanodots inside an antidot matrix of the opposite material were investigated by micromagnetic simulations. The results show that magnetization reversal of Fe nanodots can be made more reliable and predictable by surrounding them with a Ni matrix. Oppositely, Ni nanodots in an Fe matrix do not seem to be technologically useful since magnetization reversal occurs via large domain walls through the whole system, located mostly inside the nickel nanodots. 

However, more investigation on different distances between the dots is necessary to study other possible magnetic states, such as horseshoe or onion states in the Fe antidot matrix surrounding the nickel nanodots.

## Data Availability

The data created in this study are fully depicted in the article.
